# Integrated cluster- and case-based surveillance for detecting stage III zoonotic pathogens: an example of Nipah virus surveillance in Bangladesh

**DOI:** 10.1017/S0950268814002635

**Published:** 2014-10-24

**Authors:** A. M. NASER, M. J. HOSSAIN, H. M. S. SAZZAD, N. HOMAIRA, E. S. GURLEY, G. PODDER, S. AFROJ, S. BANU, P. E. ROLLIN, P. DASZAK, B.-N. AHMED, M. RAHMAN, S. P. LUBY

**Affiliations:** 1 International Centre for Diarrhoeal Disease Research, Bangladesh (icddr,b), Dhaka, Bangladesh; 2 Centers for Disease Control and Prevention (CDC), Atlanta, GA, USA; 3 EcoHealth Alliance, New York, NY, USA; 4 Directorate General of Health Services, Ministry of Health and Family Welfare, Dhaka, Bangladesh; 5 Institute of Epidemiology Disease Control and Research (IEDCR), Dhaka, Bangladesh; 6 Stanford University, Stanford, CA, USA

**Keywords:** Cluster, encephalitis, Nipah, outbreak, stage III pathogen, surveillance

## Abstract

This paper explores the utility of cluster- and case-based surveillance established in government hospitals in Bangladesh to detect Nipah virus, a stage III zoonotic pathogen. Physicians listed meningo-encephalitis cases in the 10 surveillance hospitals and identified a cluster when ⩾2 cases who lived within 30 min walking distance of one another developed symptoms within 3 weeks of each other. Physicians collected blood samples from the clustered cases. As part of case-based surveillance, blood was collected from all listed meningo-encephalitis cases in three hospitals during the Nipah season (January–March). An investigation team visited clustered cases’ communities to collect epidemiological information and blood from the living cases. We tested serum using Nipah-specific IgM ELISA. Up to September 2011, in 5887 listed cases, we identified 62 clusters comprising 176 encephalitis cases. We collected blood from 127 of these cases. In 10 clusters, we identified a total of 62 Nipah cases: 18 laboratory-confirmed and 34 probable. We identified person-to-person transmission of Nipah virus in four clusters. From case-based surveillance, we identified 23 (4%) Nipah cases. Faced with thousands of encephalitis cases, integrated cluster surveillance allows targeted deployment of investigative resources to detect outbreaks by stage III zoonotic pathogens in resource-limited settings.

## INTRODUCTION

Stage III zoonotic pathogens spill over from animals to humans, but because their basic reproduction number (*R*_0_) is <1, they produce only stuttering chains of person-to-person transmission that terminate [1]. Nevertheless, stage III zoonotic pathogens represent a risk for human pandemics, because the transmissibility of individual strains varies and each chain of transmission provides an opportunity for evolutionary adaptation of the pathogen to human hosts [2–4]. Some stage III zoonotic pathogens including Nipah virus (NiV), influenza virus and Middle East respiratory syndrome coronavirus have a high human fatality rate [4–7]. If one strain of stage III pathogens with a high fatality rate acquires the capacity of efficient person-to-person transmissibility (*R*_0_ > 1), it may produce a devastating global pandemic [2]. Effective surveillance for pandemic threats would permit early identification of potentially pandemic strains, monitor the change in viral transmissibility between humans [8] and support early initiation of public health responses. Focusing surveillance on clusters of cases that are linked temporally and geographically and so are more likely to represent person-to-person transmission is potentially an efficient strategy for deploying limited surveillance resources to identify pandemic threats. Nevertheless, a single surveillance strategy may not always be adequate to achieve multiple purposes and therefore, integration of multiple surveillance strategies may be more effective. There are limited examples of such integration for the stage III pathogens.

NiV is a stage III zoonotic pathogen that has caused recognized fatal outbreaks in Bangladesh almost every year since 2001 [9–11]. Between 2001 and 2007, 87% of the identified Nipah cases in Bangladesh died [7]. Drinking raw date palm sap contaminated with bat saliva or urine that contains NiV is the main transmission route between *Pteropus* bat, the reservoir host of NiV, and the population in Bangladesh [7]. In many identified Nipah outbreaks in Bangladesh, we found no evidence of person-to-person transmission. Nevertheless, in several outbreaks people who came in direct contact with secretions of Nipah cases, also became infected through person-to-person transmission [10, 12, 13].

In 2006, the Institute of Epidemiology, Disease Control and Research (IEDCR) of the Government of Bangladesh, with the collaboration of the International Centre for Diarrhoeal Disease Research, Bangladesh (icddr,b) introduced year-round surveillance focused on identification of encephalitis clusters in 10 government hospitals and subsequent investigation of identified clusters. The objective was to identify encephalitis outbreaks including NiV at an early stage, to understand the risk factors and transmission pathways, and to introduce timely public health interventions to contain the identified outbreaks.

Since the identified NiV outbreaks in Bangladesh had all occurred during January–March, IEDCR and icddr,b also expanded surveillance activities specifically during this Nipah transmission season to screen all individual meningo-encephalitis cases admitted in three surveillance hospitals for NiV, even when they were not part of a cluster [7]. We designated this expansion of surveillance as case-based surveillance.

This paper describes the performance of cluster-based surveillance in detecting stage III zoonotic pathogens, explains features of cluster-based surveillance to monitor their person-to-person transmissibility and explores the added benefits of nested case-based surveillance, considering NiV as an example.

## METHODS

### Cluster-based surveillance

#### Surveillance sites

**Fig. 1.**
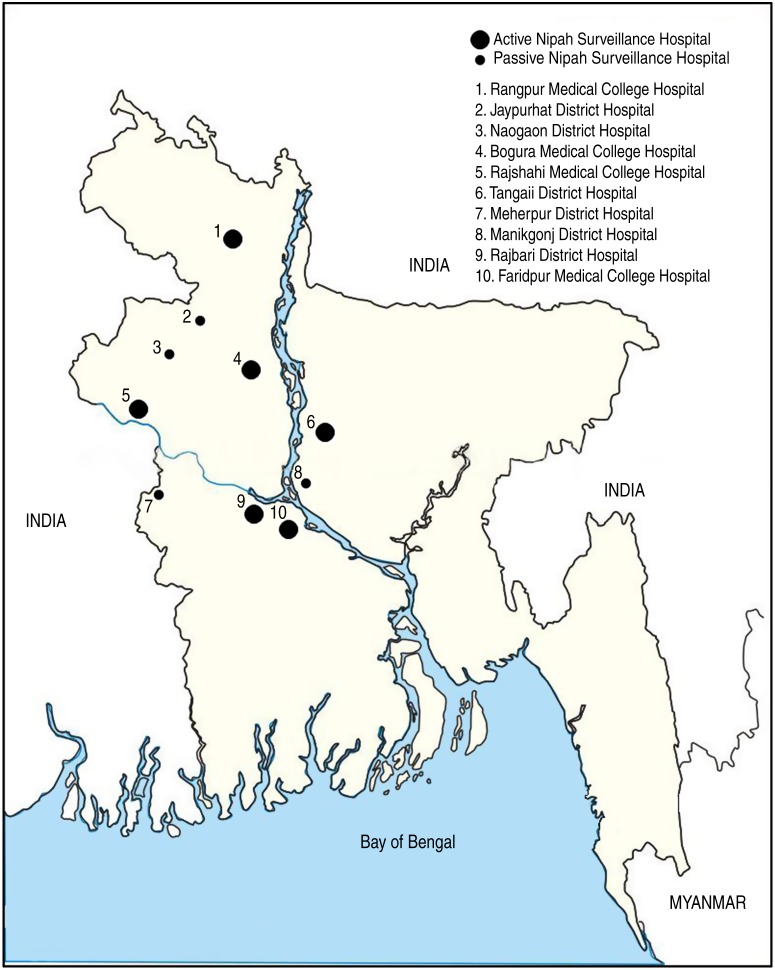
Location of 10 surveillance hospitals in northwest and central Bangladesh.

In February 2006, IEDCR and icddr,b established active surveillance in three tertiary and seven district-level government hospitals in northwest and central Bangladesh (Fig. 1). In February 2007, four district hospitals were converted to passive surveillance sites because few encephalitis patients were admitted to those hospitals. Passive sites only reported to IEDCR and icddr,b if they identified an unusually large number of meningo-encephalitis cases. Surveillance physicians in the remaining six active sites reported clusters as soon as they were identified and the total number of encephalitis cases admitted monthly.

#### Activities in surveillance hospitals and cluster identification

Surveillance physicians listed admitted patients in paediatric and adult medicine wards with suspected meningo-encephalitis, defined as fever (axillary temperature >38·5 °C) with recent altered mental status or seizure or other neurological deficits suggestive of either diffuse or localized brain injury. Surveillance physicians recorded detailed addresses and phone numbers of listed cases and identified if they were from the same community. In their communities in order to identify additional encephalitis, physicians asked admitted cases and/or their caregivers about other sick persons or recent deaths with similar symptoms who did not report to the hospitals or died at their initial stage of illness. We defined a cluster as ⩾2 meningo-encephalitis cases aged ⩾5 years living within 30 min walking distance of each other who developed illness within 3 weeks of one another. In rural Bangladesh villagers are quite knowledgeable about the major events occurring in other households within a village due to strong prevailing kinship [14] and it usually requires <30 min to walk across a village or workplace. The cut-off of 3 weeks was chosen by adding the incubation period of NiV (1–10 days) and period of transmissibility (6–11 days) [15]. If physicians identified an admitted meningo-encephalitis case with ⩾1 sick persons with similar symptoms reported in his/her community, they also considered that case as part of a cluster. After identification of a cluster, surveillance physicians collected illness and exposure histories from cluster cases and/or their family members and blood from the living cases.

#### Cluster investigation

When a cluster was identified, an investigation team from IEDCR and icddr,b visited cases in the hospitals and/or communities to determine the cluster size and collect detailed epidemiological exposures using a standard questionnaire. The time interval between initial presentation of cases’ illnesses and visits of the investigation team to hospitals and/or communities varied; therefore, many acute cases had either died or their illness resolved by the time of investigation. The team identified family members, relatives, neighbours, friends and colleagues as the proxy respondents for the dead or severely ill cases who were unable to respond. The team also asked the cases and/or their family members, relatives, neighbours, friends and colleagues of the cases and the community leaders to identify if there were other persons with fever and altered mental status or convulsion, or recent deaths with similar symptoms in their community. The objective of this investigation was to assess whether cases within an observed cluster shared an exposure or if they were clustered by chance. To establish epidemiological linkages, the team explored whether clustered cases lived together, were family members, resided in the same village, or worked together; they investigated common exposures such as drinking date palm sap, contact with the sick person with similar illness, or contact with sick animals. They also matched the exposure dates and illness onset dates. When we identified NiV clusters with epidemiological linkages, we considered whether clusters were most likely formed due to person-to-person transmission of NiV or due to multiple NiV introductions from a common exposure to date palm sap. In addition, the team collected blood from living cases in hospitals or communities.

#### Laboratory procedures

The field team centrifuged blood and transported specimens to IEDCR on wet ice or liquid nitrogen. Before 2007, specimens were shipped to the US Centers for Disease Control and Prevention (CDC) to test for Nipah-specific IgM by enzyme immunoassay. Since 2007, IEDCR and icddr,b have tested specimens using a Nipah-specific IgM ELISA provided by CDC, which has a high sensitivity, specificity and concordance with other Nipah-specific ELISAs [16]. CDC retested all positive samples and every tenth negative sample for reconfirmation and quality control.

### Case-based Nipah surveillance

During the NiV transmission season (January–March), we collected blood and CSF of all listed meningo-encephalitis cases from Faridpur and Rajshahi Medical College Hospitals from 2007 and from Rangpur Medical College Hospital from 2008 to find all NiV cases presenting to these hospitals in addition to cluster-based surveillance. These three tertiary hospitals were chosen for case-based surveillance because large numbers of Nipah cases had been identified previously from the catchment areas of these hospitals.

### Definition of Nipah case-patients

*Confirmed Nipah case.* We defined a laboratory-confirmed NiV case as a meningo-encephalitis case with detectable serum Nipah IgM antibody.

*Probable Nipah case.* We defined a probable case as a meningo-encephalitis case who had epidemiological linkages with a confirmed NiV case within 3 weeks’ of their illness but whose blood was not collected due to death or the first blood sample was negative and second sample was not collected due to death.

*Isolated Nipah case.* We defined an isolated NiV case as a meningo-encephalitis case with detectable serum Nipah IgM antibody but who was not part of a cluster or who had no epidemiological linkages with any other known probable or confirmed NiV cases even if they were part of a cluster.

### Data analysis

We categorized the clusters based on cluster size and calculated the proportion of clusters where Nipah cases were identified. We conducted *χ*^2^ analysis to determine whether a cluster size of >3 was associated with presence of Nipah cases within an identified cluster.

Because of the public health importance of identifying strains of NiV with an increased capacity for person-to-person transmission, for the NiV clusters with person-to-person transmission, we estimated *R*_0_ using the outbreak's size or total number of individuals infected by the primary case to monitor the human transmissibility of the different NiV outbreaks and to detect if there were any possible differences in transmissibility which could serve to highlight strain differences [17, 18]. By definition, *R*_0_ measures the average number of cases infected by a single primary infectious case. Therefore, one primary Nipah case will produce *R* secondary Nipah cases in the first generation [19]. Each of these secondary cases will again infect *R* persons, producing a total of *R* × *R* = *R*^2^ cases in the second generation, *R*^3^ cases in third generation and so on. Hence, the total outbreak size (*A*) can be expressed by the following geometric progression: *A* = 1 + *R* + *R*^2^ + *R*^3^ + …, where 1 is the primary case. For stuttering outbreaks when *R*_0_ < 1, the expression is reduced to *A* = 1/(1 – *R*), which means if *R* = 0·9, the total outbreak size will be 10 (one primary case and nine is the summation of secondary, tertiary, etc. generation cases) [18]. Since outbreak size *A* is known from detailed cluster investigation, we estimated *R*_0_ as 1–1/*A*, from the reduced expression.

We calculated and compared proportions of NiV yield by both cluster surveillance and case-based surveillance. For cluster-based surveillance, we calculated NiV yield as Nipah cases presenting to hospitals in clusters plus Nipah cases identified in the communities or referred to hospitals by the investigation team. For case-based surveillance, we calculated NiV yield as sporadic Nipah cases presenting to three surveillance hospitals.

### Ethical approval

Healthy participants or legal guardians of the participants provided informed consent for participation. The Ethical Review Committee of icddr,b reviewed and approved the protocol for NiV surveillance and outbreak investigation.

## RESULTS

### Cluster-based surveillance

Between February 2006 and September 2011, surveillance physicians listed 5887 encephalitis cases in 10 surveillance hospitals. In these cases, physicians identified 62 clusters. A total of 176 (3%) cases were included in these 62 clusters; 147 were identified in hospitals while 29 were identified from communities. The median age of the clustered cases was 21 years (interquartile range 7–40); 120 (68%) cases were male. Seventy-four (42%) cases died (Table 1). At least one patient died in 34 (55%) clusters.

**Table 1.** Demographics, fatality and Nipah status of the clustered encephalitis cases identified by cluster-based surveillance, 2007–2011 (N = 176).

Characteristics	Number of cases, *n*/*N* (%)
Age, years	
⩽10	62/176 (35%)
11–20	24/176 (14%)
21–40	52/176 (29%)
41–60	30/176 (17%)
⩾61	8/176 (5%)
Male	120/176 (68%)
Fatality	74/176 (42%)
Blood specimens collected	127/176 (72%)
Nipah cases	62/176 (35%)
Confirmed	28/62 (45%)
Probable	34/62 (55%)

A total of 127 (72%) samples were collected from cases identified in clusters, and a single blood specimen was collected from at least one case in each cluster. These included specimens from 87 acute and 31 convalescent cases. We collected both acute and convalescent blood samples from nine cases. In 10 (17%) of 62 identified clusters, we identified 62 Nipah cases. Of these, 32 Nipah cases presented at hospitals in clusters, while 30 were identified in communities. Twenty-eight were laboratory-confirmed Nipah cases and 34 were probable cases. The yield for NiV infections from blood samples of cluster-based surveillance was 22% (28 laboratory-confirmed NiV infections/127 blood samples tested).

**Fig. 2.**
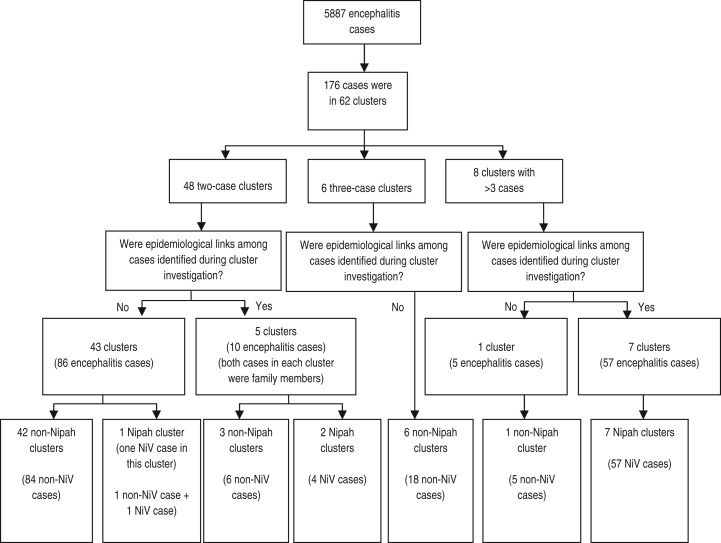
Summary of clusters identified through cluster-based surveillance, 2006–2011.

Forty-eight (78%) clusters were two-case clusters, six (10%) were three-case clusters and the remaining eight (12%) clusters had 4–20 cases (Fig. 2). In 43 two-case clusters, cases had no common exposure or apparent history of contact with each other. In only one of these 43 two-case clusters without epidemiological linkages, were we able to identify a laboratory-confirmed Nipah case. In the other five two-case clusters, both cases were from the same family. Two of these five were Nipah clusters but the other three clusters had no laboratory evidence of Nipah infection. One patient died from two of these three non-Nipah clusters. In these two clusters where one patient died, illness of both cases began on the same day in one cluster and 1 week apart in the other.

None of the three-case clusters had any epidemiological linkages or NiV infection among cases. The presence of Nipah cases within a cluster was associated with cluster size >3 (*P* < 0·001). Among the eight clusters having >3 cases, seven (88%) were Nipah clusters. We identified epidemiological linkages between cases in each of these seven clusters. We identified a total of 57 Nipah cases in these seven Nipah clusters, either laboratory-confirmed or probable. Of these 57 Nipah cases, 47 (82%) patients died. Of the seven Nipah clusters having epidemiological linkages, we identified evidence of person-to-person transmission in four clusters and multiple NiV introductions from date palm sap for three clusters. The estimated *R*_0_ for the four NiV clusters with person-to-person transmission ranged from 0·75 to 0·88 (Table 2). One cluster of five cases, which included two deaths and had no epidemiological linkages between cases, was not caused by NiV and remains undiagnosed (Fig. 2).

**Table 2.** Cluster size of seven Nipah clusters and estimated R_0_

Cluster no.	Year of identification	Cluster size (*A*)	Basic reproduction number (*R*_0_)
1	2007	7	0·86
2	2010	8	0·88
3	2011	4	0·75
4	2011	8	0·88

### Case-based Nipah surveillance

Between 2007 and 2011, we identified 982 patients meeting the meningo-encephalitis case definition at three tertiary surveillance hospitals participating in case-based surveillance. Of these patients, 173 (18%) died (Table 3). Surveillance physicians collected blood samples from 608 patients of which 23 (4%) were isolated laboratory-confirmed Nipah cases.

**Table 3.** Number of meningo-encephalitis patients identified at three tertiary hospitals through case-based surveillance during January–March, 2007–2011

Year	Rajshahi Medical College Hospital	Faridpur Medical College Hospital	Rangpur Medical College Hospital	Total (*N*)	Deaths *n* (%)	No. of sporadic Nipah virus cases
2007	56	16	—	72	18 (25%)	1
2008	69	60	66	195	38 (19%)	1
2009	135	10	84	229	13 (6%)	4
2010	82	113	57	252	52 (21%)	8
2011	55	60	119	234	52 (22%)	9
Total	397	259	326	982	173 (18%)	23

## DISCUSSION

In our setting of scarce resources with thousands of encephalitis cases, cluster-based surveillance redirects public health attention away from pathogens having no demonstrated person-to-person transmissibility and towards specimens of spatio-temporally clustered cases that more likely represent stage III zoonotic pathogens and pandemic threats. Ninety-seven per cent of the listed encephalitis cases were excluded from specimen testing for stage III zoonotic pathogens. Importantly, community-level investigations allowed cluster-based surveillance to distinguish clusters with epidemiological linkages from clusters without epidemiological linkages. Out of the 62 clusters, we identified epidemiological linkages in 12 clusters of which nine were Nipah clusters. Because of this efficiency, cluster-based surveillance has been used in other settings [20, 21].

Our integrated surveillance data explain different modes of introduction of stage III zoonotic pathogens into human populations. We identified sporadic NiV introductions from the case-based surveillance, and two types of Nipah clusters or outbreaks with a high fatality rate from cluster-based surveillance. One type of Nipah outbreak occurred as a result of multiple introductions of NiV to humans; another type occurred due to person-to-person transmission of NiV. Analyses of the viral strains identified by cluster- and case-based surveillance permit monitoring changes of viral factors including person-to-person transmission of such deadly pathogenic agents. We identified four small short-lived Nipah chains of transmission due to person-to-person transmission that had *R*_0_ for NiV ranging from 0·75 to 0·88. The estimates of *R*_0_ for the strains causing person-to-person transmission were higher than our earlier estimates of the average *R*_0_ for NiV in Bangladesh [7]. This is consistent with the presumed heterogeneity of capacity for person-to-person transmission among stage III zoonotic agents [22]. Furthermore, NiV strains identified from case-based surveillance or Nipah clusters with multiple introductions from date palm sap, had no person-to-person transmission and can be used as a comparison group to understand transmission heterogeneity and to explain why these infections failed to spread [23]. In addition to viral factors, community-level cluster investigations and in-depth assessment of exposures also help us to identify host risk behaviours associated with increased person-to-person transmission. These insights can guide the implementation of strategies to reduce the spread of infections during epidemics or pandemics. For instance, community investigations have permitted development of acute interventions to reduce exposures during outbreaks, and have contributed to the development of broader longer term intervention strategies to reduce exposures [24–26].

This study has some limitations. We did not explore aetiological agents for non-Nipah clustered cases. The 114 non-Nipah cases were in three categories. First, 107 non-Nipah cases from 49 non-Nipah clusters that had no epidemiological linkages between each other. These were likely caused by common pathogens with limited pandemic potential [27, 28]. Second, six non-Nipah cases from three two-case non-Nipah clusters that had epidemiological linkages between cases. Temporal analysis of the symptom onset of the cases’ in these clusters suggests that cases likely had co-infections or were infected by one or more non-Nipah agents that had limited person-to-person transmission capability. Since cases died in these clusters, they are a priority for further diagnostic attention. Collecting and testing specimens from such non-Nipah clustered cases with epidemiological linkages as a part of a rapid response could be considered as an appropriate investment of limited resources to identify or discover pathogens. Third, a single non-Nipah case from a two-case Nipah cluster where we did not identify epidemiological linkage. We believe this cluster was formed by chance and the non-Nipah cases were infected by a common agent causing encephalitis.

In Bangladesh, as in other low-income countries, aetiological diagnosis is not performed as part of clinical evaluation of encephalitis cases; therefore, testing of specimens for stage III pathogens is not routine. Indeed, the cost for aetiological diagnosis of stage III pathogens is high and may make surveillance prohibitively expensive in low-resource settings if such surveillance requires that all encephalitis cases are tested. Individual case-based Nipah surveillance was a supplemental approach of cluster-based surveillance to identify isolated NiV introductions that would have been missed by the cluster approach. Rational use of resources for additional testing of all encephalitis cases only during the Nipah transmission season and only in limited hospitals where the greatest number of encephalitis cases were admitted, provided supplemental information about isolated introductions of stage III zoonoses and improved the comprehensiveness of surveillance. We were able to establish a targeted case-based surveillance for NiV due to its known epidemiology, including seasonal transmission period, in addition to the year-round cluster-based surveillance that could provide information for multiple stage III pathogens having similar clinical presentations. However, for unknown stage III pathogens, lack of epidemiological information may limit the opportunity for targeted case-based surveillance.

Our surveillance had limited coverage with 10 government hospitals. We probably missed some clusters because some of the cases might have been admitted to other hospitals. For instance, this surveillance activity missed one Nipah outbreak in 2007 because patients were admitted to a government hospital that was not a surveillance hospital [12]. Many meningo-encephalitis cases also might not have been hospitalized due to the low healthcare utilization in Bangladesh [29]. In addition, surveillance physicians might have failed to list some patients, which might have left some clusters undetected [10]. Physicians’ assessment of whether two cases were of 30 min walking distance from each other may occasionally be inaccurate because they do not have precise geographical knowledge about the catchment areas of hospitals. We could not collect specimens due to death of many clustered cases. This is another inherent limitation of a cluster-based approach for infections with a high case-fatality rate like NiV because of the time lag between when initial cases are hospitalized and the cluster is identified. Surveillance would be more effective if we could prioritize the rapid cluster identification and deployment of a field investigation team capable of collecting various diagnostic specimens including post-mortem biopsies [30]. In addition, delays between recognition of a cluster and deployment of a field investigation team limit our capability to diagnose the early cases of NiV in a cluster and to respond with prevention activities to contain the spread of infection. Our calculation of *R*_0_ risks generating a biased estimate of the mean transmissibility of all NiV strains. However, the primary objective of this surveillance is specifically to identify agents with unusually high person-to-person transmission, rather than estimating a general mean. All these limitations reduce the sensitivity of cluster-based surveillance; however, given the severe resource constraints in low-income settings where emerging pathogens pose the greatest threat, the integration of surveillance strategies provides a practical strategy for optimally focusing limited resources on identifying stage III zoonotic pathogens.

Researchers have identified regions characterized as emerging disease ‘hotspots’ where pathogens are more likely to emerge [31]. Different stage III pathogens with different case-fatality rates and different socio-cultural settings and health systems may require different approaches and integration. Integration of cluster-based surveillance with other surveillance strategies could be evaluated in other low-income settings in emerging disease hotspots. Resources for surveillance in the highest risk areas of emerging infections remain scarce. Strategies to reduce cost have the potential to widen the global surveillance net for identifying and monitoring pathogens at highest risk for causing a severe human pandemic.
